# Supplementary material for: Airway Bacterial Biodiversity in Exhaled Breath Condensates of Asthmatic Children—Does It Differ from the Healthy Ones?
Source: J Clin Med. 2022 Nov 16;11(22):6774. doi: 10.3390/jcm11226774 (PMC9698396; doi:10.3390/jcm11226774)
Supplement: Supplementary file 1 [file jcm-11-06774-s001.zip › jcm-2027444-supplementary.pdf]

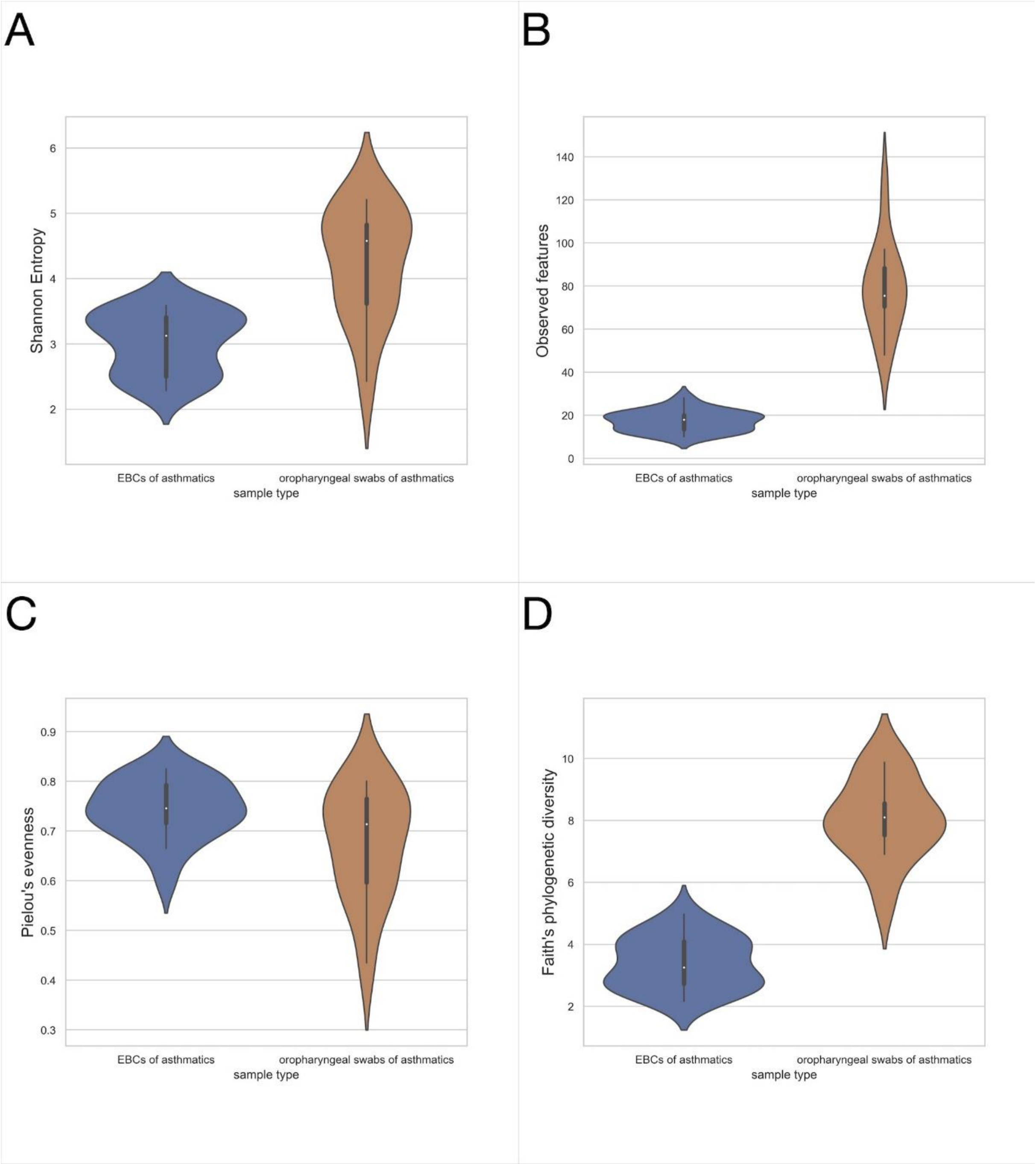

Supplementary Figure S1. Alpha-diversity metrics for sample type of asthmatic patients. (A) Shannon diversity index, mean 4.226 (±0.846) vs. 3.029 (±0.462), p= 1.907×10^{-4}; (B) Observed Features, mean 78.6 (±21.0) vs. 17.4 (±4.8), p= 3.651×10^{-6}; (C) Pielou's evenness, mean 0.672 (±0.111) vs. mean 0.742 (±0.060), p= 0.068; (D) Faith's Phylogenetic Diversity, mean 7.982 (±1.263) vs. 3.370 (±0.807), p= 3.777^{-6}, swab (n=12) vs. EBC (n=19) respectively, Kruskal-Wallis test.

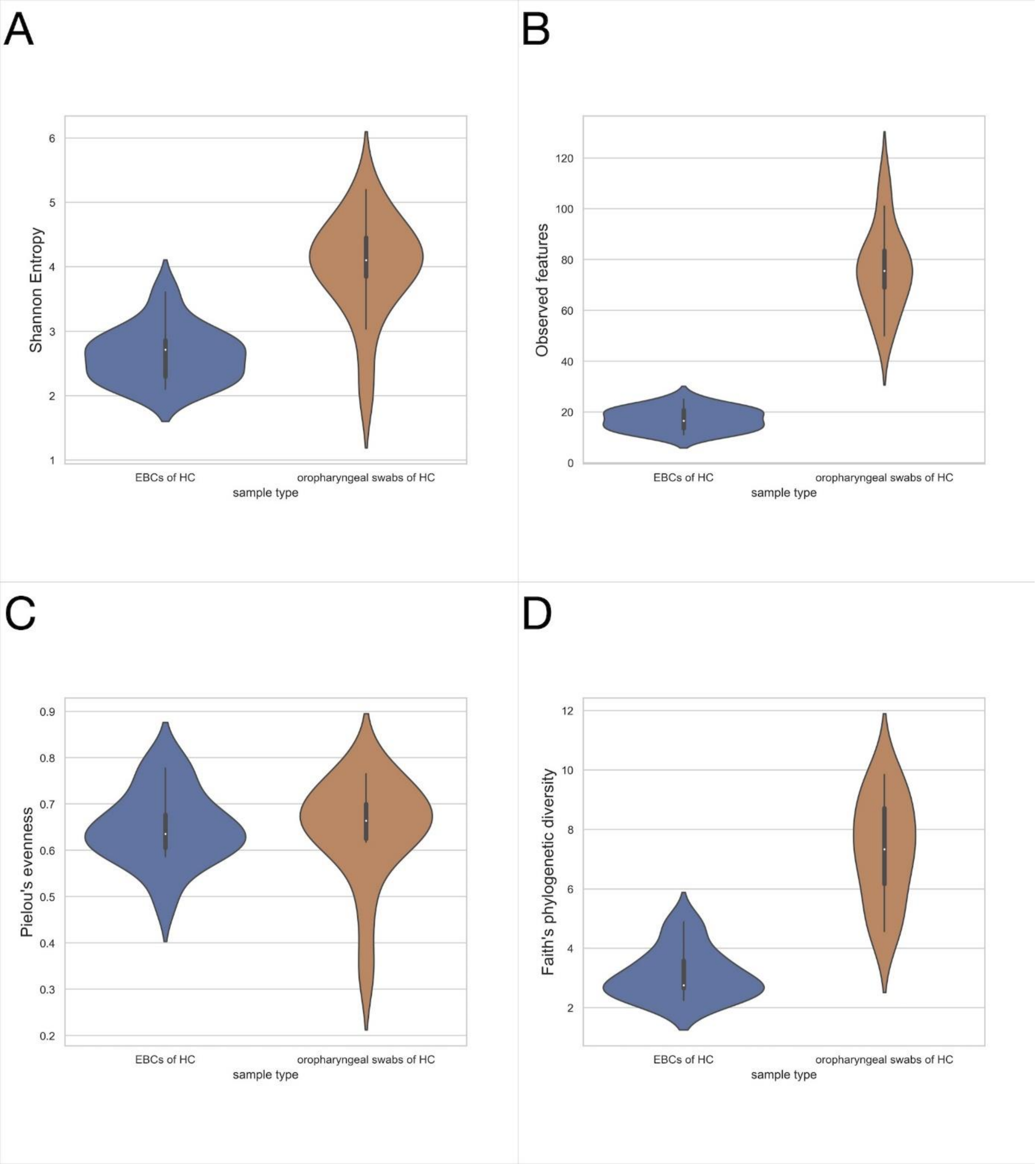

Supplementary Figure S2. Alpha-diversity metrics for sample type of healthy controls (HC). (A) Shannon diversity index, mean 4.018 (±0.763) vs. 2.642 (±0.424), $p=1.648\times10^{-4}$; (B) Observed Features, mean 76.4 (±16.6) vs. 17.4 (±4.4), $p=6.607\times10^{-6}$; (C) Pielou's evenness, mean 0.644 (±0.110) vs. mean 0.648 (±0.078), p=0.520; (D) Faith's Phylogenetic Diversity, mean 7.542 (±1.730) vs. 3.150 (±0.839), $p= 1.927\times10^{-5}$, swab (n=14) vs. EBC (n=14) respectively, Kruskal-Wallis test.

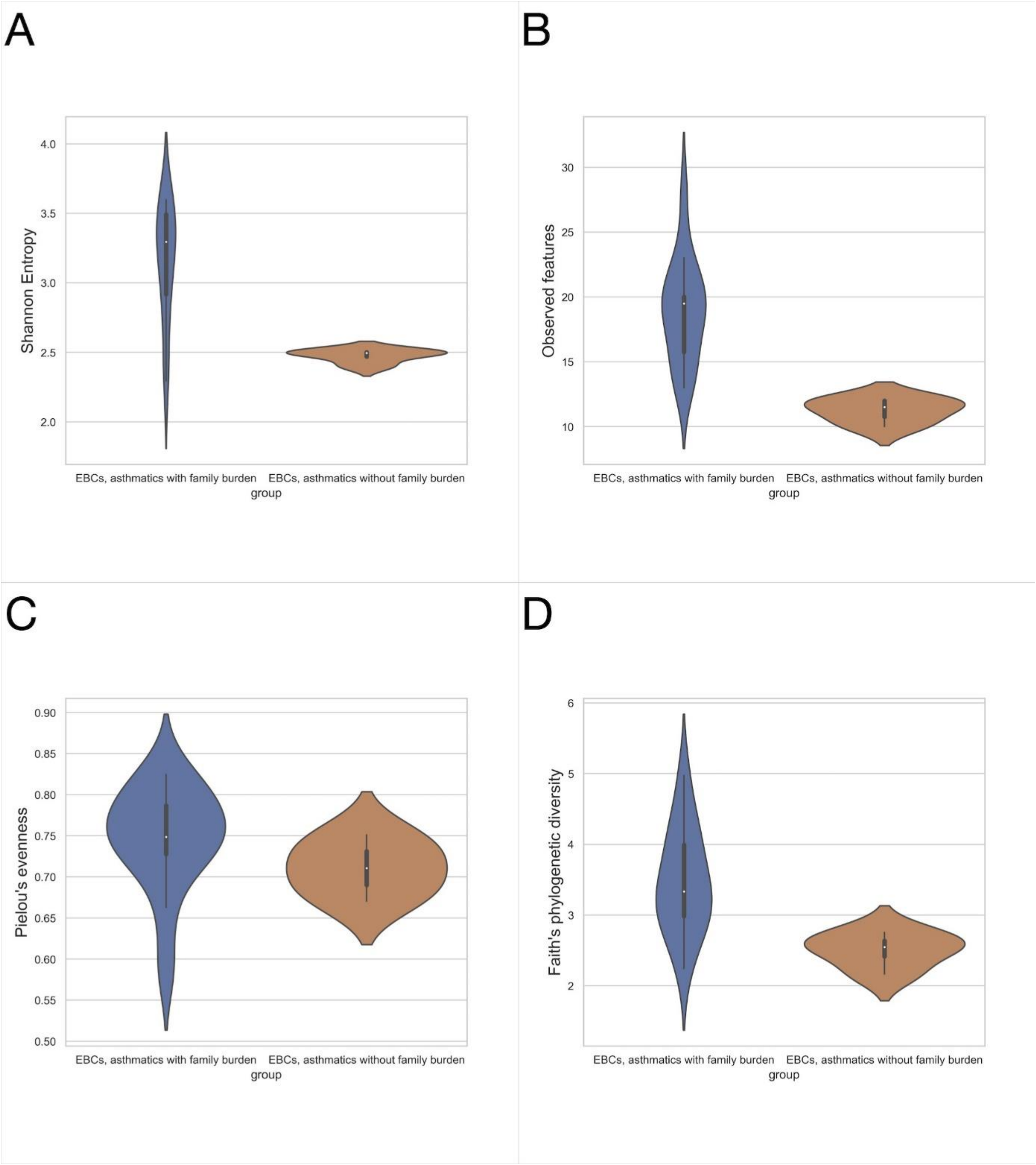

Supplementary Figure S3. Alpha-diversity metrics for EBCs of asthmatics with family burden (n=14) vs. without family burden of allergic diseases (n=4), mean respectively: (A) Shannon diversity index, 3.158 (±0.412) vs. 2.476 (±0.048), p=0.026; (B) Observed Features, 18.929 (±3.97) vs. 11.25 (±0.957), p=0.003; (C) Pielou's evenness, 0.747 (±0.063) vs. 0.711 (±0.035), p=0.243; (D) Faith's Phylogenetic Diversity, 3.47 (±0.737) vs. 2.506 (±0.249), p=0.011, Kruskal-Wallis test.

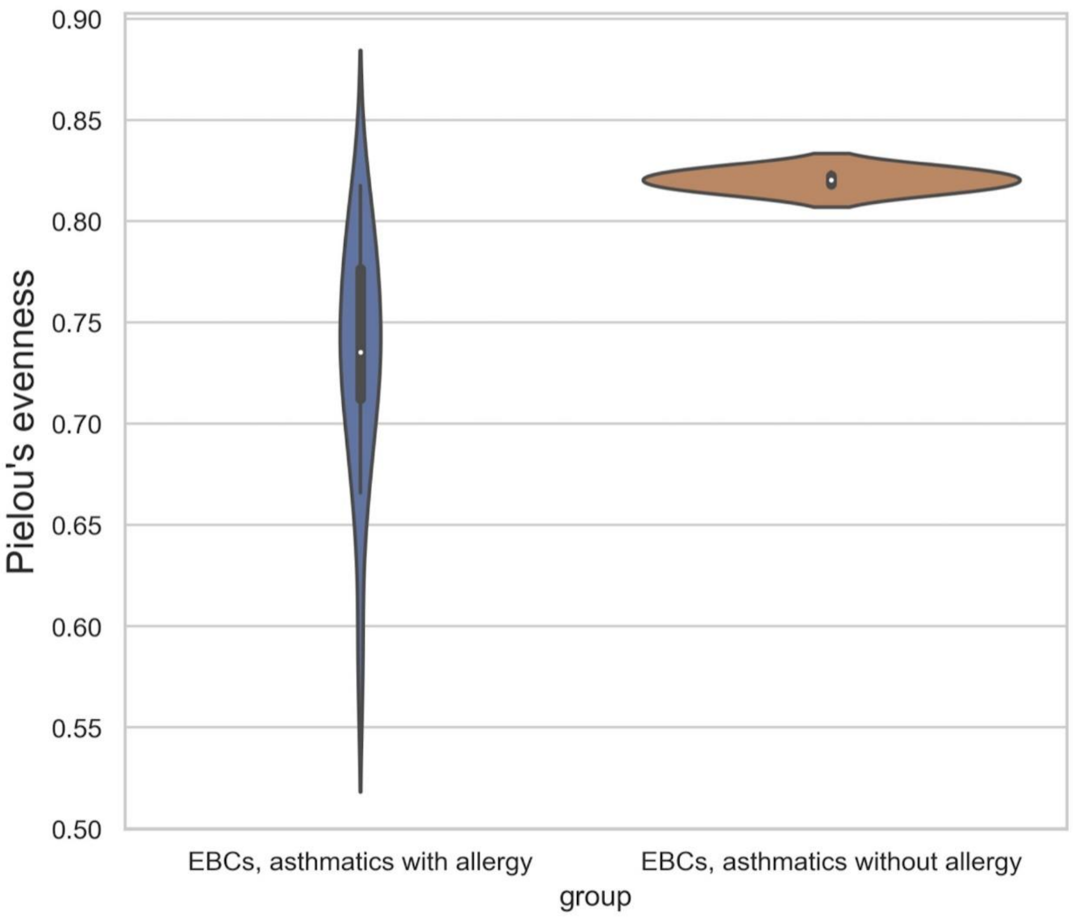

Supplementary Figure S4. Alpha-diversity, Pielou's evenness, asthmatic patients with concurrent inhalant allergies (n=17) vs. asthmatic patients without allergies (n=2), 0.734 (±0.056) vs. 0.821 (±0.006), p=0.034, Kruskal-Wallis test.

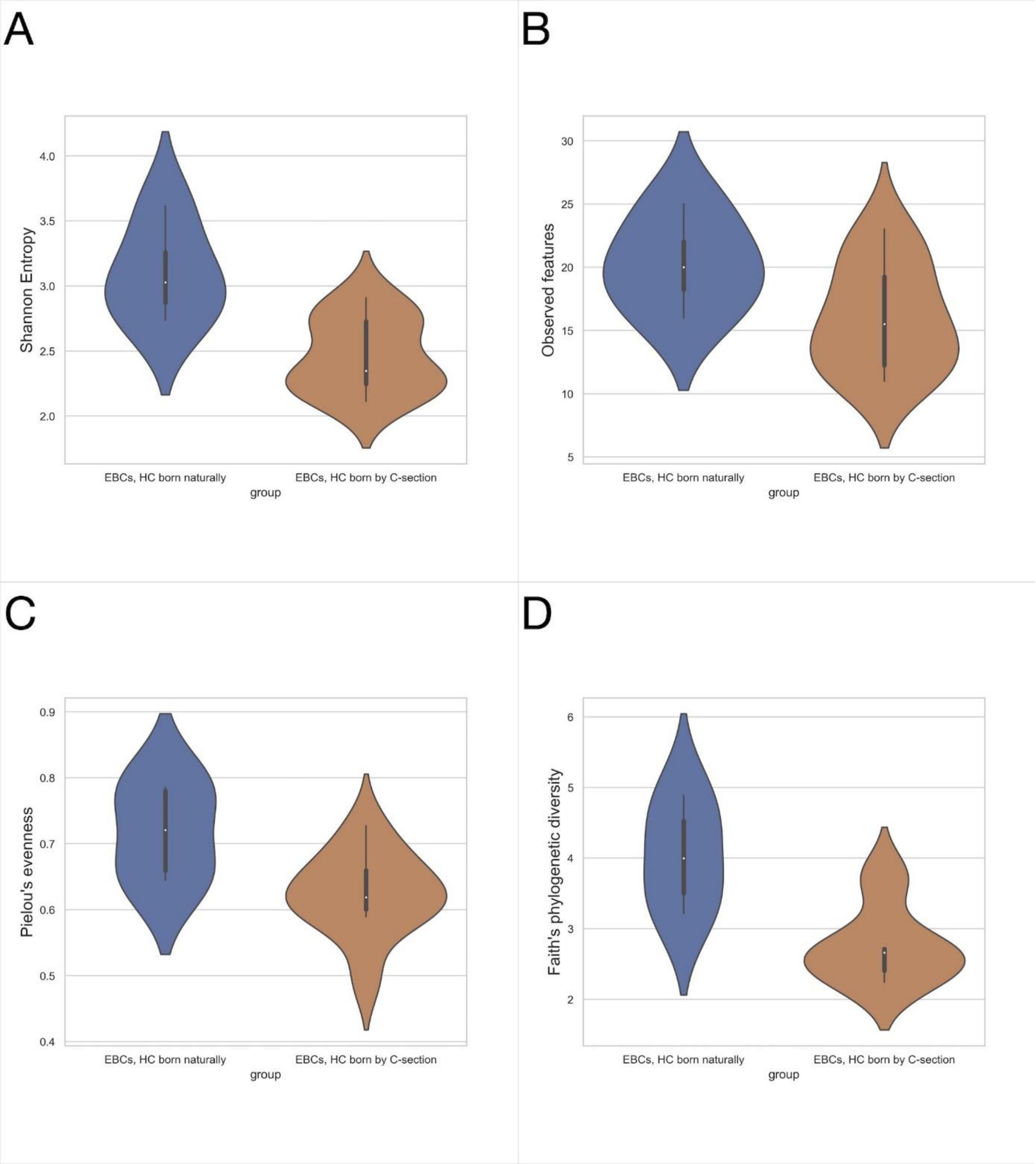

Supplementary Figure S5. Alpha-diversity metrics for EBC, healthy controls born naturally (n=4) vs. born by C-section (n=10). (A) Shannon diversity index, mean Shannon, mean 3.102 (±0.378) vs. 2.461 (±0.284), p=0.011; (B) Observed features mean 20.3 (±3.8) vs. 16.0 (±4.190), p=0.087; (C) Pielou's evenness, mean 0.718 (±0.074) vs. 0.624 (±0.062), p=0.090; (D) Faith's Phylogenetic Diversity, mean 4.027 (±0.76) vs. 2.764 (±0.537), p=0.034; Kruskal-Wallis test.
